# The Royal Orthopædic Hospital

**Published:** 1904-07-02

**Authors:** Tate S. Mansford

**Affiliations:** Secretary. Royal Orthopædic Hospital, 55 Bolsover Street, W.


### EDITOR'S LETTER-BOX.

[Our Correspondents are reminded that prolixity is a great bar to publication, and that brevity of style and conciseness of statement greatly facilitate early insertion.]

### THE ROYAL ORTHOPÆDIC HOSPITAL.

*To the Editor of* THE HOSPITAL.

SIR,—I am instructed by the Committee of the Royal Orthopædic Hospital to inform you with reference to the circular issued by the secretary of the City Orthopædic Hospital, dated January 14th, 1904, that the statement contained in that circular to the effect that the Royal Orthopædic Hospital has ceased to exist, is entirely untrue. As you have been informed, the Royal Orthopædic Hospital is amalgamating with the National Orthopædic Hospital under the sanction and with the strong approval of King Edward's Hospital Fund for London.

Arrangements have been made for the erection of a new building in which the work of the combined institution will be carried on under one management. In the meantime the work of the Royal Orthopædic Hospital is being carried on in temporary premises at 55 Bolsover Street, W., where both in-patients and out-patients continue to be received.

So far as my Committee can learn, not one patient of the Royal Orthopædic Hospital has gone to the City Orthopædic Hospital, and the suggestion that the amalgamation has in any way increased the work of the City Orthopædic Hospital is without foundation.

Your obedient servant,
TATE S. MANSFORD, Secretary.
Royal Orthopædic Hospital, 55 Bolsover Street, W.

